# Supplementary material for: Identification of photoperiod-induced specific miRNAs in the adrenal glands of Sunite sheep (Ovis aries)
Source: Front Vet Sci. 2022 Jul 22;9:888207. doi: 10.3389/fvets.2022.888207 (PMC9354845; doi:10.3389/fvets.2022.888207)
Supplement: Supplementary file 1 [file Data_Sheet_1.ZIP › Supplementary file/miRDeep2 file.zip!/miRDeep2 file/Novel_Structure/SP42A2/Novel_189.pdf]

Provisional ID	:	NC_019475.2_32883
Score total	:	143.9
Score for star read(s)	:	3.9
Score for read counts	:	136.2
Score for mfe	:	2.2
Score for randfold	:	1.6
Score for cons. seed	:	
Total read count	:	279
Mature read count	:	270
Loop read count	:	0
Star read count	:	9

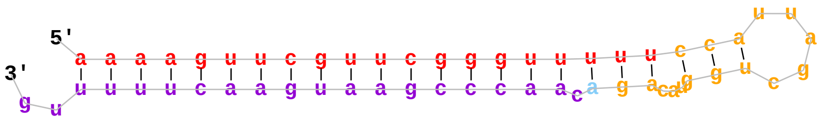

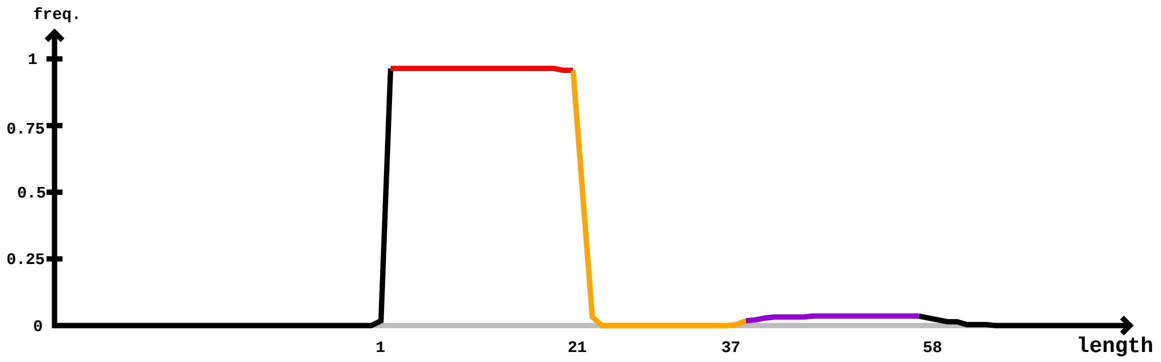


```
                                  Mature                          Star
5'- ccuggggaggaagggauguacuggguuggcccaaaaguucguucggguuuuuccauuagcugguacagacaacccgaaugaacuuuuuggccaauucaauauuuccagc  -3'   obs
    ccuggggaggaagggauguacuggguuggcccaaaaguucguucggguuuuuccauuagcugguacagacaacccgaaugaacuuuuuggccaauucaauauuuccagc        exp
    (((.....)))...(((.(((.(((((((((.(((((((((((((((((((((((.....)))...))).))))))))))))))))).))))))))))).))).)))...   reads     mm      sample
    ...............................caaaaguucguucggguuuuu.........................................................   2         0       seq
    ...............................Aaaaaguucguucggguuuuuc........................................................   1         1       seq
    ...............................caaaaguucguucggguuuuuc........................................................   2         0       seq
    ................................aaaaguucguucggguuu...........................................................   2         0       seq
    ................................aaaaguucguucggguuuuC.........................................................   20        1       seq
    ................................aaaaguucguucggguuuuu.........................................................   146       0       seq
    ................................aaaaguucguucggguuuuuc........................................................   88        0       seq
    ................................aaaaguucguucggguuuuucU.......................................................   7         1       seq
    ................................aaaaguucguucggguuuuucc.......................................................   2         0       seq
    ....................................................................acaacccAaaugaacuuuuug....................   1         1       seq
    .....................................................................caacccgaaugaacuuuuug....................   4         0       seq
    ......................................................................aacccgaaugaacuuuuuU....................   1         1       seq
    .......................................................................acccgaaugaacuuuuuggcc.................   2         0       seq
    ........................................................................cccgaaugaacuuuuuggcA.................   1         1       seq
    ............................................................................aaCgaacuuuuuggccaau..............   1         1       seq
```
